# Successful blind lung isolation with the use of a novel double-lumen endobronchial tube in a patient undergoing lung transplantation with massive pulmonary secretion: A case report

**DOI:** 10.1097/MD.0000000000016869

**Published:** 2019-08-16

**Authors:** Yijun Seo, Namo Kim, Hyo Chae Paik, Dahee Park, Young Jun Oh

**Affiliations:** a Department of Anesthesiology and Pain Medicine; b Anesthesia and Pain Research Institute; c Department of Thoracic and Cardiovascular Surgery, Yonsei University College of Medicine, Seoul, South Korea.

**Keywords:** airway management, idiopathic pulmonary fibrosis, lung transplantation, one-lung ventilation

## Abstract

**Rationale:**

Precise lung isolation technique with visual confirmation is essential for thoracic surgeries to create a safe and clear surgical field. However, in certain situations, such as when patients have massive pulmonary secretion or when the fiberoptic bronchoscopy (FOB) is not applicable, lung isolation has been performed blindly.

**Patient concern:**

A 52-year-old woman, whose airway was unable to visualize with FOB due to massive pulmonary secretion, was presented for bilateral sequential lung transplantation. Extracorporeal membranous oxygenation, tracheostomy, and mechanical ventilation were applied to the patient for 39 days preoperatively as a bridge for lung transplantation.

**Diagnosis:**

Patient was diagnosed with an idiopathic pulmonary fibrosis and obesity.

**Intervention:**

Initially, height-based blind positioning with a conventional double-lumen endobronchial tube (DLT) failed to ventilate the patient properly, and the confirmation of DLT positioning with FOB was impossible due to massive pulmonary secretion. Therefore, a novel DLT (ANKOR DLT) that has one more cuff, located at a point between the distal opening of the tracheal lumen and the starting point of bronchial cuff, than conventional DLT was used for the lung isolation in the patient.

**Outcomes:**

After the completion of lung graft, FOB finding showed that the ANKOR DLT was optimally positioned at the tracheobronchial tree of the patient, and its depth was 2.5 cm shallower than that of the conventional tube.

**Lessons:**

ANKOR DLT would be a feasible choice to achieve successful blind lung isolation when the use of FOB is impossible to achieve the optimal lung isolation.

## 1 Introduction

Precise lung isolation technique is essential for thoracic surgeries to create a safe and clear surgical field. Ideal positioning of double-lumen endobronchial lumen (DLT) has been confirmed by fiberoptic bronchoscope (FOB).^[1,2]^ Alternatively, blind lung isolation technique, such as the lung compliance,^[3]^ bronchial cuff pressure change,^[4]^ or height-based assumption,^[5]^ has been used when FOB is unavailable or not applicable in certain situations.

Here, we report the successful blind lung isolation by using a novel DLT which has one more cuff than the conventional DLT, developed by one of the authors, in a patient undergoing lung transplantation with massive pulmonary secretion. This would be the first report describing the clinical use of a novel DLT in the literature. Written informed consent for the publication of the clinical details and clinical image were obtained from the patient postoperatively.

## 2 Case report

**Figure 1:**
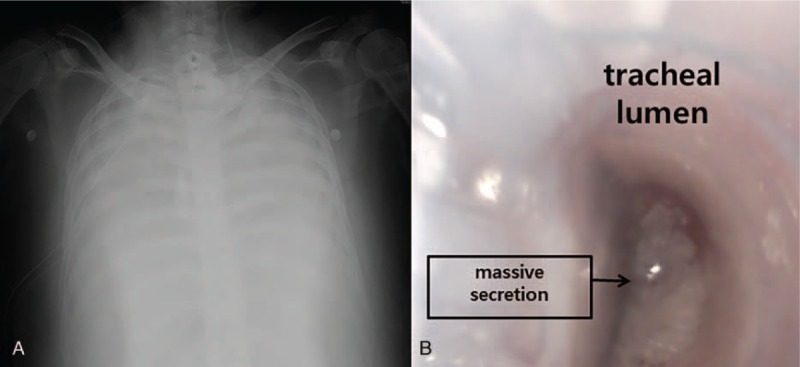
Perioperative images of the patient. A, Preoperative chest x-ray, showing severe diffuse lung consodiation. B, Bronchoscopic finding, showing massive pulmonary secretion within the conventional double-lumen endobronchial tube.

A 52-year-old woman (height 159 cm, weight 94 kg) with idiopathic pulmonary fibrosis and obesity was presented for bilateral sequential lung transplantation with right lung graft first under extracorporeal membrane oxygenation (ECMO). Preoperative pulmonary function test (%, predicted) showed forced expiratory volume in 1 second, 0.88 L (37%); forced vital capacity in 1 second, 0.92 L (30%); forced expiratory volume in 1 second/forced vital capacity, 95%; and diffusing capacity of carbon monoxide, 8.3 L/mmHg/min (28%). Veno-venous ECMO, tracheostomy, and mechanical ventilation were applied to the patient for 39 days preoperatively as a bridge for lung transplantation. Preoperative chest radiography showed severe diffuse lung consolidations (Fig. 1A). Preoperative cardiac echocardiography showed reduced right ventricle systolic function, mild pulmonary hypertension (right ventricular systolic pressure 47 mmHg), D-shaped left ventricle with slightly decreased left ventricle systolic function (ejection fraction 56%).

**Figure 2:**
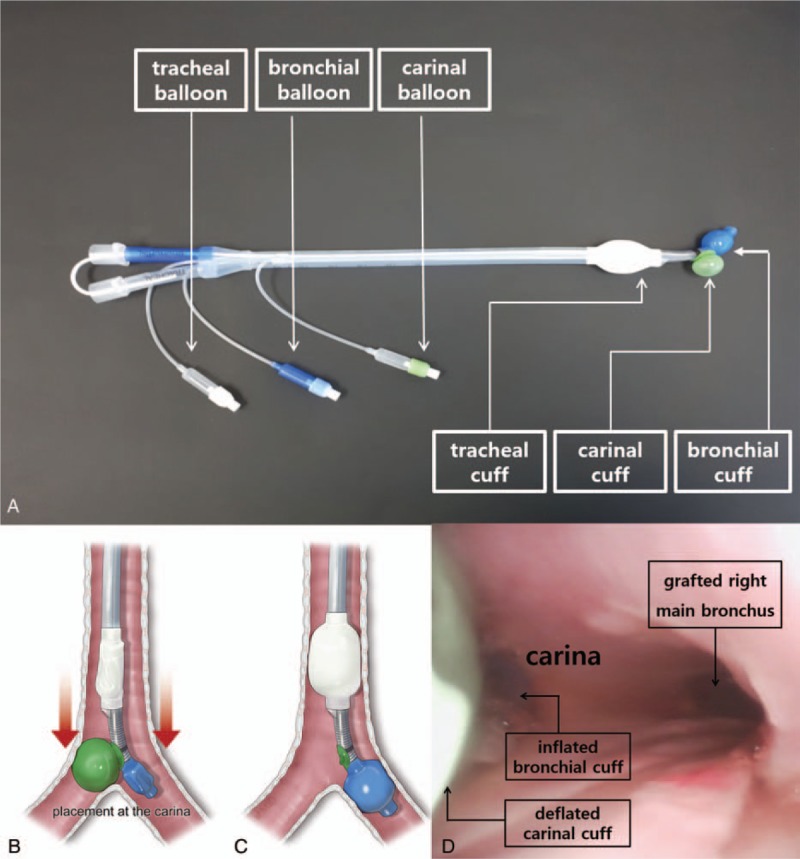
The design of a novel double-lumen endobronchial tube (ANKOR DLT) and its application to the patient. A, Compared with conventional DLT, ANKOR DLT has one more cuff, “carinal cuff,” that is located at a point between the distal opening of the tracheal lumen and the starting point of bronchial cuff. B, Once the carinal cuff of the tube passed through the vocal cord of the patient, it was turned to the left, and carinal cuff was inflated with 6 mL of air. It was transiently supposed to form an inverted “Y” shape with the inflated carinal cuff and the distal part of bronchial lumen of the tube, which functionally anchored the tube at the keel-shaped carinal ridge. C, After the deflation of the carinal cuff, the tracheal cuff and the bronchial cuff of the tube were inflated with 5 and 2 mL of air, respectively. D, After the completion of right lung graft, bronchoscopic finding showed that the tube was properly positioned in the tracheobronchial tree of the patient showing the upper margin of the bronchial cuff was slightly seen at between the carina and the left main bronchial orifice without the obstruction of the tracheal lumen.

The anesthesia was induced with midazolam 3.0 mg, sufentanil 50 μg, and rocuronium 50 mg. Initially, a 35 Fr left-sided DLT (Shiley, Covidien, Mansfield, MA) was inserted via oral route for lung isolation and blindly placed at 28.5 cm depth at the level of upper incisor of the patient according to the height-based blind positioning of DLT.^[5]^ However, both lungs were unable to ventilate, and the confirmation for the positioning of DLT with FOB (FIVE 4.0, Karl Storz, Tuttlingen, Germany) was impossible due to massive pulmonary secretion (Fig. 1B). Despite the effort to suck out the pulmonary secretion with the suction catheter and FOB, any anatomic structures of tracheobronchial tree were invisible. We decided to use a new DLT (ANKOR, Insung Med, Wonju, South Korea) of left-sided 35 Fr (Fig. 2A). After the carinal cuff of the tube passed through the vocal cord of the patient, the tube was turned to left side, and carinal cuff was inflated with 6 mL of air (Fig. 2B). The tube was advanced further toward left main bronchus, and it stopped at 26.0 cm of depth at the level of upper incisor of the patient. After the deflation of the carinal cuff, the tracheal cuff and the bronchial cuff of the tube were inflated with 5 and 2 mL of air, respectively (Fig. 2C). After the completion of right lung graft, bronchoscopic finding showed that the tube was properly positioned at the tracheobronchial tree of the patient showing the upper margin of the bronchial cuff was slightly seen at between the carina and the left main bronchial orifice without the obstruction of the tracheal lumen (Fig. 2D).^[6]^ Surgeons indicated that the use of the new DLT provided excellent operating conditions during both lung grafts. The operation time was 10 hours and 50 minutes under veno-arterial ECMO. The patient was transferred to intensive care unit with veno-venous ECMO. ECMO was applied for postoperative day (POD) 13, and the patient was moved to the general ward at POD 37.

## 3 Discussion

One-lung ventilation is usually achieved by exact lung isolation technique with DLT or bronchial blocker (BB).^[7]^ Ideal positioning of DLT or BB is usually confirmed by FOB.^[1,2]^ DLT is most commonly used to achieve lung isolation for lung transplantation. However, using DLT requires the anesthesiologist's proficiency and preference. The DLT has been described as “difficult tubes” because of an increased rigidity and their large outside diameter.^[8]^ Moreover, 39% of anesthesiologists with limited thoracic anesthesia experience were unable to achieve successful lung separation regardless of the type of lung isolation device due to poor knowledge of endoscopic bronchial anatomy.^[9]^ It requires also normal anatomy of patient and confirmation of bronchoscopy. Blind positioning of DLT is sometimes used in a difficult airway patient or condition, confirmed by using the compliance of both lungs or prescribed formula (12.5 + [0.1 × height] cm).^[4]^ However, the success rate of blind lung isolation technique, done by dedicated thoracic anesthesiologists, was only 63%.^[1]^ However, if the patients have end-stage lung disease combined with a massive pulmonary secretion, bronchoscopic confirmation of exact lung isolation with the use of FOB or the blind lung isolation with lung compliance would be impossible.

The ANKOR DLT was developed to provide the simple method of lung isolation for thoracic surgery. Compared with the conventional DLT, it has one more cuff that is located at a point between the distal opening of the tracheal lumen and the starting point of bronchial cuff. The carinal cuff was designed to expand toward the opposite side of bronchial lumen of the tube. Consequently, it is transiently supposed to form an inverted “Y” shape with the inflated carinal cuff and the distal part of bronchial lumen of the tube, which functionally anchors the tube at the keel-shaped carinal ridge. Once the ANKOR DLT stops at some depth of tracheobronchial tree, that point is considered as an ideal depth of DLT positioning for lung isolation. Afterwards, the deflation of the carinal cuff of the ANKOR DLT renders the tube function as a conventional DLT. Therefore, as shown in this case, the use of ANKOR DLT would help the anesthesiologists to perform the more successful lung isolation technique which usually takes about 1 minute as long as the structure of main tracheobronchial tree of patients were intact, even in the patients with an end-stage lung disease combined with massive pulmonary secretion. In this case, minimizing the time wasting for lung isolation might have been crucial for the viable donor lungs during the surgery, although ECMO applied to the patient preoperatively might have provided oxygen to the patient in this case. Therefore, we expect that this new DLT would work well in patients who have severe destructive lung parenchyma, hemothorax, or for anesthesiologists with limited experience in lung isolation.

In conclusion, the ANKOR DLT would be a feasible choice to achieve successful blind lung isolation when the use of fiberoptic bronchoscopy is impossible to achieve the precise lung isolation as shown in this case report. Further well-designed clinical studies are required for its effectiveness on blind lung isolation in various clinical circumstances.

## Acknowledgments

The authors thank MID (Medical Illustration & Design), a part of the Medical Research Support Services of Yonsei University College of Medicine, for all artistic support related to this work.

## Author contributions

**Conceptualization:** Hyo Chae Paik, Young Jun Oh.

**Data curation:** Namo Kim.

**Investigation:** Dahee Park.

**Resources:** Yijun Seo.

**Supervision:** Young Jun Oh.

**Validation:** Dahee Park.

**Visualization:** Namo Kim, Hyo Chae Paik.

**Writing – original draft:** Yijun Seo.

**Writing – review & editing:** Young Jun Oh.
